# The systematic comparison between Gaussian mirror and Model-X knockoff models

**DOI:** 10.1038/s41598-023-32605-5

**Published:** 2023-04-04

**Authors:** Shuai Chen, Ziqi Li, Long Liu, Yalu Wen

**Affiliations:** 1 Department of Health Statistics, School of Public Health, Shanxi Medical University, No 56 Xinjian South Road, Yingze District, Taiyuan, Shanxi Province China; 2 Department of Statistics, University of Auckland, 38 Princes Street, Auckland Central, Auckland, New Zealand 1010

**Keywords:** High-throughput screening, Statistical methods, Genomics

## Abstract

While the high-dimensional biological data have provided unprecedented data resources for the identification of biomarkers, consensus is still lacking on how to best analyze them. The recently developed Gaussian mirror (GM) and Model-X (MX) knockoff-based methods have much related model assumptions, which makes them appealing for the detection of new biomarkers. However, there are no guidelines for their practical use. In this research, we systematically compared the performance of MX-based and GM methods, where the impacts of the distribution of explanatory variables, their relatedness and the signal-to-noise ratio were evaluated. MX with knockoff generated using the second-order approximates (MX-SO) has the best performance as compared to other MX-based methods. MX-SO and GM have similar levels of power and computational speed under most of the simulations, but GM is more robust in the control of false discovery rate (FDR). In particular, MX-SO can only control the FDR well when there are weak correlations among explanatory variables and the sample size is at least moderate. On the contrary, GM can have the desired FDR as long as explanatory variables are not highly correlated. We further used GM and MX-based methods to detect biomarkers that are associated with the Alzheimer’s disease-related PET-imaging trait and the Parkinson’s disease-related T-tau of cerebrospinal fluid. We found that MX-based and GM methods are both powerful for the analysis of big biological data. Although genes selected from MX-based methods are more similar as compared to those from the GM method, both MX-based and GM methods can identify the well-known disease-associated genes for each disease. While MX-based methods can have a slightly higher power than that of the GM method, it is less robust, especially for data with small sample sizes, unknown distributions, and high correlations.

## Introduction

The identification of biomarkers that are informative for diagnosis, prognosis and treatment of complex diseases is a critical step towards precision medicine, an emerging model of healthcare that delivers treatments tailored according to individual’s profiles. The high-dimensional biological data (e.g. genomics, transcriptomics and proteomics) have provided ample data resources for such purposes, and their analyses have made multiple testing a routine^1–3^.

The most classic methods to correct for multiple testing focus on controlling the family-wise error rate (FWER) that is defined as the probability of obtaining at least one false discovery. The Bonferroni correction^4,5^, Holm’s step-down^6^ and Hochberg’s step-up procedures^7^ as well as some of the latest developments have all been used to control the FWER. Despite their popularity, producing no false positives with a high probability can lead to a substantial degree of conservativeness, reducing the power to detect true positives. The trade-off between type I error and power becomes an important issue for high-dimensional data, where thousands or even millions of tests are simultaneously conducted in the hope of screening out important biomarkers worthy of further investigations. The false discovery rate (FDR)^8^, defined as the expected proportion of discoveries that are false positive, is a more recently proposed metric for controlling multiplicity. Its goal is to find discoveries with the proportion of type I error controlled at a specified level (e.g. 0.1). It usually has higher power to detect disease-associated markers as compared to FWER-based methods. For high-dimensional biological data, researchers are willing to substantially increase the total amount of findings at the expense of accepting a small proportion of false positives, and FDR becomes the dominant criterion^9–15^. Therefore, for the rest of this paper, we focus on methods that aim at controlling the FDR.

The most classic and population FDR control methods are constructed based on p-values (e.g. Benjamini and Hochberg step-up procedure (BH) and Storey’s q-value method^16^). They generally require reliable p-values that are independent or have special restrictions on their dependence structures^17–22^. However, in practice, reliable p-values could be hard to obtain partially due to limited sample sizes and/or questionable distributional assumptions and the correlations among variables can be high and of arbitrary types (e.g. co-expressed genes^23–28^), limiting the application of p-value-based FDR methods. Barber and Candès^29^ have recently proposed the novel knockoff filter method that imposes much relaxed assumptions than the classical FDR methods. Their basic idea is to evaluate the importance of each variable by comparing its importance with its knockoffs that are guaranteed to have no effects on the outcomes, and thus it does not require the derivation of valid p-values. Indeed, Barber and Candès have shown that their knockoff method can achieve theoretical FDR control as long as the sample size is larger than the number of variables ($n > p$), and it has similar level of performance as the BH procedure with p-values well calibrated. For high-dimensional data ($n< p$), Candès et al.^30^ proposed the Model-X knockoff, an extension of the original knockoff method. Rather than treating each explanatory variable as fixed^29^, the Model-X knockoff method considers them as random variables and makes inference through utilizing their joint distribution that is assumed to be perfectly known. The Model-X knockoff neither requires reliable p-values nor independence among variables, making it a much more flexible framework for the FDR control. While promising, knockoff-based methods heavily depend on the construction of valid knockoffs, which can be challenging in practice when the conditional distributions among explanatory variables are unknown^31^. Although Barber et al*.*^32^ showed that knockoff methods are robust against the assumptions of the joint distribution and the inflation in the false positives only depends on the errors in the estimation of the conditional distribution of each variable, it still can be very challenging in the estimation of the distributions for high-dimensional data^33^.

To alleviate the challenges embedded in constructing valid knockoffs, Xing X et al.^33^ proposed the Gaussian mirror method, which requires no knowledge of the joint distribution of explanatory variables and can theoretically control the FDR. Like knockoff-based methods, Gaussian mirror does not rely on the assumptions of valid p-values and their independence. For each explanatory variable, Gaussian mirror creates a pair of mirror variables by plus or minus a random perturbation to the original observed value. It constructs its test statistics by comparing the sum and difference of regression coefficients of the two mirror variables, and chooses a scaler such that the test statistics are symmetric under the null hypotheses. It can be applied to variables that follow flexible distributions (e.g. genomic and transcriptomic count data), and it can also consider non-linear effects when combined with neural networks^34^. While Gaussian mirror method is flexible, its power heavily depends on the conditional independence among the mirror variables, which is a necessary condition for valid test statistics.

While the newly developed Gaussian mirror and Model-X knockoff models are both promising in the analysis of high-dimensional biological data, each of them has their own advantages and disadvantages. For example, the performance of knockoff model highly depends on how accurate the knockoff can be constructed, whereas the performance of Gaussian mirror relies on whether the conditional independence can be established easily. While both can be used in practice, due to their recent and concurrent development, consensus is lacking on which methods should be used for a particular analysis. To bridge this gap, we systematically compared their performance under various simulation settings, including different distributions of explanatory variables, different amount of non-relevant variables, and different levels of correlations among explanatory variables. We applied them to detect biomarkers that are associated with Alzheimer’s disease-related traits and further compared their consistencies and discrepancies. In the following sessions, we briefly overviewed the technical details of each model, and then conducted simulation studies and analyzed the genomic data obtained from Alzheimer’s Disease Neuroimaging Initiative to compare their performance. Finally, we summarized our main findings and provided practical recommendations.

## Methods

As our main purpose of this study is to provide systematic comparisons between the Model-X knockoff (MX)^30^ and the Gaussian mirror (GM) methods^33^, we first briefly provide the technical details for these two methods in the following sessions.

### The Gaussian mirror method

The GM method creates a pair of "mirror variables" $\left({x}_{j}^{+},{x}_{j}^{-}\right)$ for each variable ${x}_{j}$: ${x}_{j}^{+}={x}_{j}+{c}_{j}{z}_{j}$ and ${x}_{j}^{-}={x}_{j}-{c}_{j}{z}_{j}$, where ${z}_{j}\sim N\left(0,{I}_{n}\right)$ and ${c}_{j}$ is a scalar. The GM method fits a regression model to obtain the coefficients for both mirror variables (respectively denoted as ${\beta }_{j}^{+}$ and ${\beta }_{j}^{-}$ for ${x}_{j}^{+}$ and ${x}_{j}^{-}$), where ordinal least squares and Lasso are used for low-dimension ($p <n$) and high-dimension ($n\le p$) settings, respectively. It defines its test statistics for ${x}_{j}$ as ${M}_{j}=\left|{\widehat{\beta }}_{j}^{+}+{\widehat{\beta }}_{j}^{-}\right|-\left|{\widehat{\beta }}_{j}^{+}-{\widehat{\beta }}_{j}^{-}\right|$, and chooses ${c}_{j}$ such that $cov\left({\widehat{\beta }}_{j}^{+}+{\widehat{\beta }}_{j}^{-},{\widehat{\beta }}_{j}^{+}-{\widehat{\beta }}_{j}^{-}\right)=0$, which is a necessary condition to guarantee the test statistics are symmetric under the null. GM regards ${x}_{j}$ as “significant” when ${M}_{j}\ge {T}_{q}$ with ${T}_{q}$ being defined as:

$${{\varvec{T}}}_{{\varvec{q}}}=\underset{{\varvec{t}}}{{\varvec{min}}}\{\widehat{{\varvec{F}}{\varvec{D}}{\varvec{P}}}\left({\varvec{t}}\right)\le {\varvec{q}}\},\,\boldsymbol{ }{\varvec{w}}{\varvec{h}}{\varvec{e}}{\varvec{r}}{\varvec{e}}\,\boldsymbol{ }\widehat{{\varvec{F}}{\varvec{D}}{\varvec{P}}}\left({\varvec{t}}\right)\triangleq \frac{\#\left\{{\varvec{j}}|{{\varvec{M}}}_{{\varvec{j}}}\le -{\varvec{t}}\right\}}{\#\left\{{\varvec{j}}|{{\varvec{M}}}_{{\varvec{j}}}\ge {\varvec{t}}\right\}\vee 1}$$

The basic rationale for the GM test is that if ${x}_{j}$ is not relevant, then ${\widehat{\beta }}_{j}^{+}$ and ${\widehat{\beta }}_{j}^{-}$ are close to zero. Therefore, the sum (i.e. $\left|{\widehat{\beta }}_{j}^{+}+{\widehat{\beta }}_{j}^{-}\right|)$ and difference (i.e. $\left|{\widehat{\beta }}_{j}^{+}-{\widehat{\beta }}_{j}^{-}\right|$) between regression coefficients approaching 0, leading to ${E(M}_{j})=0$. On contrary, if ${x}_{j}$ is associated with the outcomes, then both ${\widehat{\beta }}_{j}^{+}$ and ${\widehat{\beta }}_{j}^{-}$ are dominated by the true effect size of ${x}_{j}$. As a consequence, $\left|{\widehat{\beta }}_{j}^{+}+{\widehat{\beta }}_{j}^{-}\right|$ is much larger than $\left|{\widehat{\beta }}_{j}^{+}-{\widehat{\beta }}_{j}^{-}\right|$, leading to ${M}_{j}>0.$ Therefore, the further ${M}_{j}$ is from 0, the more important of variable can be.

### The Model-X knockoff method

The MX method first constructs a knockoff variable ${\widetilde{x}}_{j}$ for each ${x}_{j}$, where the knockoff variable $\widetilde{X}$ satisfies the following two properties: (1) exchangeability, i.e. ${\left(X,\widetilde{X}\right)}_{sawp\left(S\right)}\genfrac{}{}{0pt}{}{d}{=}\left(X,\widetilde{X}\right)$ for any subset $S \subset \{1,\dots ,p\}$; and (2) $\widetilde{X}$ is conditionally independent of the outcome given $X$ (i.e. $\widetilde{X} \perp Y|X$). A Lasso model is fitted with both $X$ and $\widetilde{X}$ as its input, where the penalty parameter $\lambda$ is chosen based on cross-validation. Let ${\widehat{\beta }}_{j}\left(\lambda \right)$ and ${\widehat{\beta }}_{(j+p)}\left(\lambda \right)$ respectively denote the regression coefficients for the $j$ th variable and its knockoff. The test statistics is defined as ${W}_{j}=\left|{\widehat{\beta }}_{j}\left(\lambda \right)\right|-\left|{\widehat{\beta }}_{(j+p)}\left(\lambda \right)\right|$. Intuitively, if ${x}_{j}$ is not relevant, then ${\widehat{\beta }}_{j}\left(\lambda \right)$ and ${\widehat{\beta }}_{(j+p)}\left(\lambda \right)$ approach zero. Therefore, under the null, ${W}_{j}$ is expected to be around zero. If ${x}_{j}$ is relevant, then $|{\widehat{\beta }}_{j}\left(\lambda \right)|$ is expected to be larger than ${\widehat{|\beta }}_{(j+p)}\left(\lambda \right)|$. Therefore, ${W}_{j}$ is expected to be away from zero. Given a targeted FDR, ${x}_{j}$ is significant when ${W}_{j}>{T}_{q}$.

It is worth noting that the performance of MX method depends on the distributional assumptions used for the knockoff generations. Candes et al. developed the Gaussian knockoff method (denoted as MX-G) for normally distributed explanatory variables, and they also developed the second-order knockoff method (denoted as MX-SO) for variables that come from other distributions. MX-SO only assumes that the second moment exists, and it does not put any assumptions on the joint distribution. Sesia et al.^35^ developed the method to create knockoffs of genotype (denoted as MX-SNP), which is specifically designed for genomic data taking values of 0, 1, and 2. To thoroughly investigate the performance of MX, we have included all these three commonly used knockoff generation methods in our analyses.

## Results

### Simulation studies

Let $Y={\left({Y}_{1},\cdot \cdot \cdot ,{Y}_{n}\right)}^{T}\in {\mathbb{R}}^{n}$ represent the response variables, $X\in {\mathbb{R}}^{n\times p}$ denote the explanatory variables, and $\epsilon ={({\epsilon }_{1},\dots ,{\epsilon }_{n})}^{T}\in {\mathbb{R}}^{n}$ be the random noise. We simulated the outcomes under an additive model:

1 $$\begin{array}{c}y=X\beta +\epsilon \end{array}$$

where $\epsilon \sim N\left(0,{I}_{n}\right)$. We have simulated 60 non-zero coefficients ($\beta \sim N(0, {\sigma }_{1}^{2}{I}_{p})$) and set the rest be zero. We chose ${\sigma }_{1}^{2}$ such that the predictors $X$ account for 90% of the total variability in the outcome. We considered a sample size of 500 and 2,000 for all our simulation studies and generated 100 Monte Carlo replicates under each setting.

#### The impact of the distributions of explanatory variables

To evaluate the impact of distributions, we generated continuous explanatory variables based on four types of distributions, including Gaussian ($X\sim N(\mathrm{0,1})$), Uniform ($X\sim U(\mathrm{0,1})$), Poisson ($X\sim Poi(3)$), and Cauchy ($X\sim C(\mathrm{1,0})$). In addition to continuous explanatory variables, we also considered the categorical variables. As our aim is to compare MX and GM methods for the analysis of biological data, we directly obtained the categorical single nucleotide polymorphism (SNP) data from the Alzheimer’s Disease Neuroimaging Initiative, so that the minor allele frequency and linkage disequilibrium (LD) structure can closely mimic the real human genome. We set the total number of variables equal to 1,200 and only considered the case, where explanatory variables are independent. Note that for SNP data that follows the multinomial distribution, we treated SNPs with LD < 0.05 as independent. The outcomes are simulated based on 60 causal variables using Eq. (1).

We first investigated whether the FDR can be well controlled when the explanatory variables follow different distributions. Table 1 and Supplementary Table S1 listed the false discovery rate when the explanatory variables follow different distributions for sample sizes of 500 and 2000, respectively. When the explanatory variables follow Cauchy distribution, all methods can barely control the FDR rate regardless of the sample size. This is not surprising, as both the expected value and the variance of Cauchy distribution are undefined, which violates the assumptions for both MX and GM models. When the independent explanatory variables follow all other distributions considered (i.e. Gaussian, Poisson, Uniform, and multinomial), the GM method has well controlled FDR regardless of sample sizes. MX-G has well controlled FDR when explanatory variables indeed come from Gaussian distributions. However, it tends to be conservative for other distributions, especially when the sample size is small. MX-SO tends to be conservative for Uniform distribution when the sample size is small, but it performs better as sample size become larger. MX-SO method depends on reliable estimates of mean and variance, which hard to obtain for small sample sizes. MX-SNP generally has well controlled FDR rates regardless of sample sizes considered.

**Table 1:** The false discovery rate when the explanatory variables follow different distributions (n = 500).

Distributions	Model X-knockoff			GM
	Gaussian	Second-order	SNPs	
Target FDR = 0.2				
Gaussian	0.203	0.215	–	0.175
Poisson	0.000	0.212	–	0.203
Uniform	0.000	0.105	–	0.189
Cauchy	0.055	0.250	–	0.116
Multinomial	–	0.224	0.180	0.191
Target FDR = 0.1				
Gaussian	0.109	0.114	–	0.099
Poisson	0.000	0.111	–	0.115
Uniform	0.000	0.052	–	0.106
Cauchy	0.052	0.211	–	0.101
Multinomial	–	0.120	0.088	0.119

The power of each method at the FDR of 0.1 and 0.2 is summarized in Table 2 and Supplementary Table S2, respectively. The power for Cauchy distribution is not presented as none of the methods have controlled FDR. Comparing the three different methods for knockoff generations, the MX-SO that uses second-order approximates to generate knockoffs has similar or better performance than other MX methods. MX-G that constructs knockoffs using the Gaussian distribution has the best performance when explanatory variables follow Gaussian distribution, but its power dropped substantially for other distributions, especially when sample size is small (Table 2). We noticed that although MX-SNP is specifically designed for SNP, it has very similar performance to MX-SO, suggesting MX-SO can have a robust performance across a range of distributions. Comparing the GM with MX methods, GM has similar or slightly worse performance than the best knockoff models in most cases, but its performance is much more stable regardless of the distributions of the explanatory variables and the sample sizes considered. For Gaussian distributions, the knockoff models tend to perform slightly better than the GM method, regardless of sample sizes. However, for other distributions (e.g. Uniform with a sample size of 500), the GM method can outperform MX method substantially. While the power of MX method depends on valid knockoffs, the power of GM only replies on the construction of symmetric test statistics under null. Therefore, GM has robust performance against the distribution of explanatory variables, and its advantages in analyzing non-Gaussian variables can be more apparent when the sample size is small.

**Table 2:** The power when explanatory variables follow different distributions (Target FDR = 0.1).

Distributions	Model-X knockoff			GM
	Gaussian	Second-order	SNPs	
n = 500				
Gaussian	0.657	0.650	–	0.629
Poisson	0.015	0.645	–	0.639
Uniform	0.016	0.353	–	0.637
Multinomial	–	0.555	0.552	0.545
n = 2000				
Gaussian	0.860	0.868	–	0.853
Poisson	0.767	0.862	–	0.853
Uniform	0.868	0.863	–	0.852
Multinomial	–	0.809	0.817	0.799

#### The impact of the noise variables 

High-dimensional biological data usually have a large amount of noise. To evaluate their impact, we gradually increased the number of noise variables from 140 to 9940 (i.e. the total number of variables increased from 200 to 10,000). Similar to the above simulations, we considered independent explanatory variables with different types of distributions (i.e. Gaussian, Poisson, and the multinomial distributions), and simulated the responses using Eq. (1).

Supplementary Table S3 shows the impact of the number of noise variables on the FDR control, and Supplementary Figs. S1 and S2 respectively depicted the trend of FDR control at the target rate of 0.1 and 0.2. Similar to the first simulation, both MX-SO and GM can reasonably control the FDR rate, whereas MX-G tends to be conservative for non-Gaussian explanatory variables, especially when the sample size is small. In addition, we noticed that the MX-G method tends to become more conservative as the amount of noise increases.

**Figure 1:**
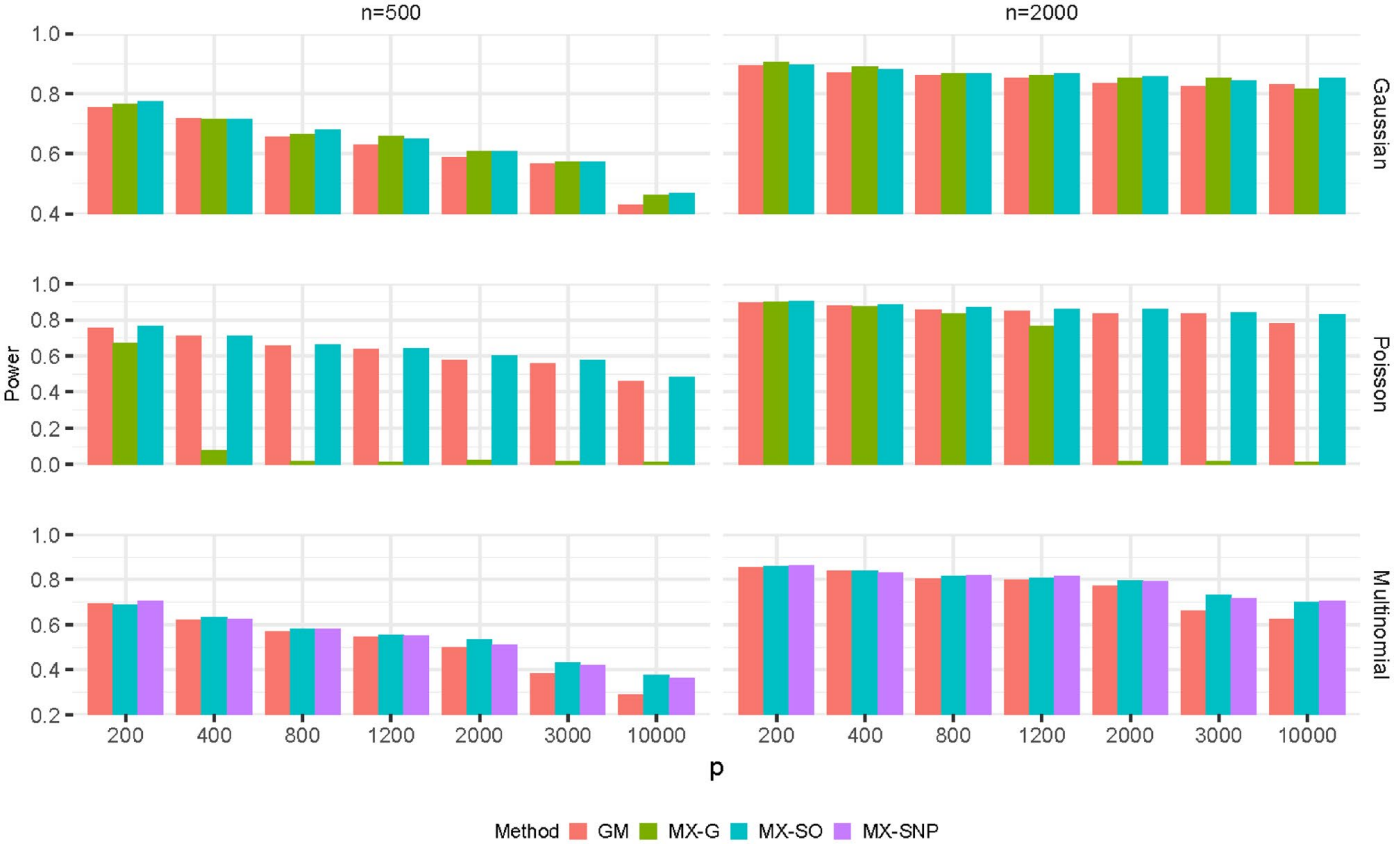
The power as the number of noise variables increases (FDR = 0.1).

Figure 1 and Supplementary Fig. S3 present the power of each method as the number of noise variables increases at the target FDR of 0.1 and 0.2, respectively. As expected, the power of each method decreases as the amount of noise increases, and the drop is more apparent when the sample size is relatively small. The amount of decrease in power is similar for all methods when the explanatory variables follow Gaussian and multinomial distributions. However, when the explanatory variables come from Poisson distribution, there is only a slightly drop in power for both MX-SO and GM methods, whereas the drop can be substantial for MX-G method. This is consistent with the FDR control level as shown in Supplementary Table S3. Regardless of the distributions and the amount of noise variables, MX-SO and GM methods tend to perform similarly.

#### The impact of correlation among explanatory variables

The MX-based method assumes that the explanatory variables are independent, and the GM method also relies on the assumption that there is only a weak correlation among them. However, for biological data, the independence assumption can be severely violated (e.g. co-expressed genes in transcriptomic data, and high LD in SNPs). Therefore, we evaluated the impact of correlations among explanatory variables in this set of simulations. Similar to the above, we considered the cases where explanatory variables follow a Gaussian, a Poisson and a multinomial distribution. To create relatedness, we set the covariance for the Gaussian and Poisson distributions to be compound symmetric with correlations ranging from 0 to 0.8. For SNP data, we varied the correlations by controlling the LD levels. When LD < 0.05, we considered the correlation between variables to be 0. For the correlation of 0.2, 0.4, 0.6 and 0.8, we controlled the LD of SNP data respectively as 0.15 ~ 0.25, 0.35 ~ 0.45, 0.55 ~ 0.65 and 0.75 ~ 0.85. We considered a total of 1140 noise variables, and simulated the outcomes using Eq. (1).

Table 3, Supplementary Table S4 show the impact of correlation among explanatory variables on the FDR control for a sample size of 500 and 2000, respectively. Supplementary Figs. S4 and S5 present the trend of the control FDR for each method as the correlation between variables increases at a target FDR of 0.1 and 0.2, respectively. In general, the GM method is the most robust method in the control of FDR rate as the correlations among explanatory variables increase. GM can control the FDR rate regardless of the correlation levels, when the sample size is large (Table S4). When the sample size is small, GM can well control the FDR rate for Gaussian variables (Table 3), but its FDR rate can be slightly inflated for non-Gaussian variables under high correlations (i.e. $\rho =0.8$). For the MX methods, when the explanatory variables follow Gaussian distribution, both MX-G and MX-SO become more conservative as the correlation increases. Under different sample sizes, the impact of correlations among Gaussian variables seems to be similar for MX-G method (i.e. the FDR is reasonably controlled when $\rho \le 0.4$), but it is more magnificent for MX-SO method under small sample sizes, which is partially due to the unstable estimates of covariance. When the explanatory variables follow Poisson distribution, the MX-G method is extremely conservative for small sample sizes (i.e. FDR $\approx 0$). However, for large sample sizes, its FDR rate seems to increase as the correlations among Poisson variables increase, although the FDR still tends to be conservative. For MX-SO, regardless of sample sizes, it becomes more conservative as the correlation among Poisson variables increases, and the impact of correlation is more apparent when sample size is small. For SNP data, the FDR increases as the correlations increase for both MX-SO and MX-SNP, and the FDR is apparently inflated when the correlation is above 0.6 regardless of sample sizes.

**Table 3:** The false discovery rate as correlations among explanatory variables increase (n = 500).

The correlation of variables	Model-X knockoff						GM		
	Gaussian		Second-order			SNPs			
	N (0, 1)	Poi (3)	N (0, 1)	Poi (3)	SNPs	SNPs	N (0, 1)	Poi (3)	SNPs
Target FDR = 0.2									
0	0.203	0.000	0.215	0.212	0.224	0.180	0.175	0.203	0.191
0.2	0.208	0.000	0.016	0.022	0.221	0.220	0.177	0.202	0.182
0.4	0.184	0.000	0.000	0.000	0.222	0.232	0.190	0.225	0.185
0.6	0.005	0.000	0.000	0.000	0.266	0.256	0.197	0.234	0.184
0.8	0.008	0.003	0.006	0.009	0.348	0.300	0.194	0.240	0.223
Target FDR = 0.1									
0	0.109	0.000	0.114	0.111	0.120	0.088	0.099	0.115	0.119
0.2	0.103	0.000	0.002	0.001	0.121	0.114	0.100	0.115	0.105
0.4	0.093	0.000	0.000	0.000	0.134	0.127	0.103	0.122	0.113
0.6	0.005	0.000	0.000	0.000	0.158	0.146	0.103	0.127	0.115
0.8	0.015	0.000	0.003	0.007	0.232	0.215	0.103	0.143	0.155

**Figure 2:**
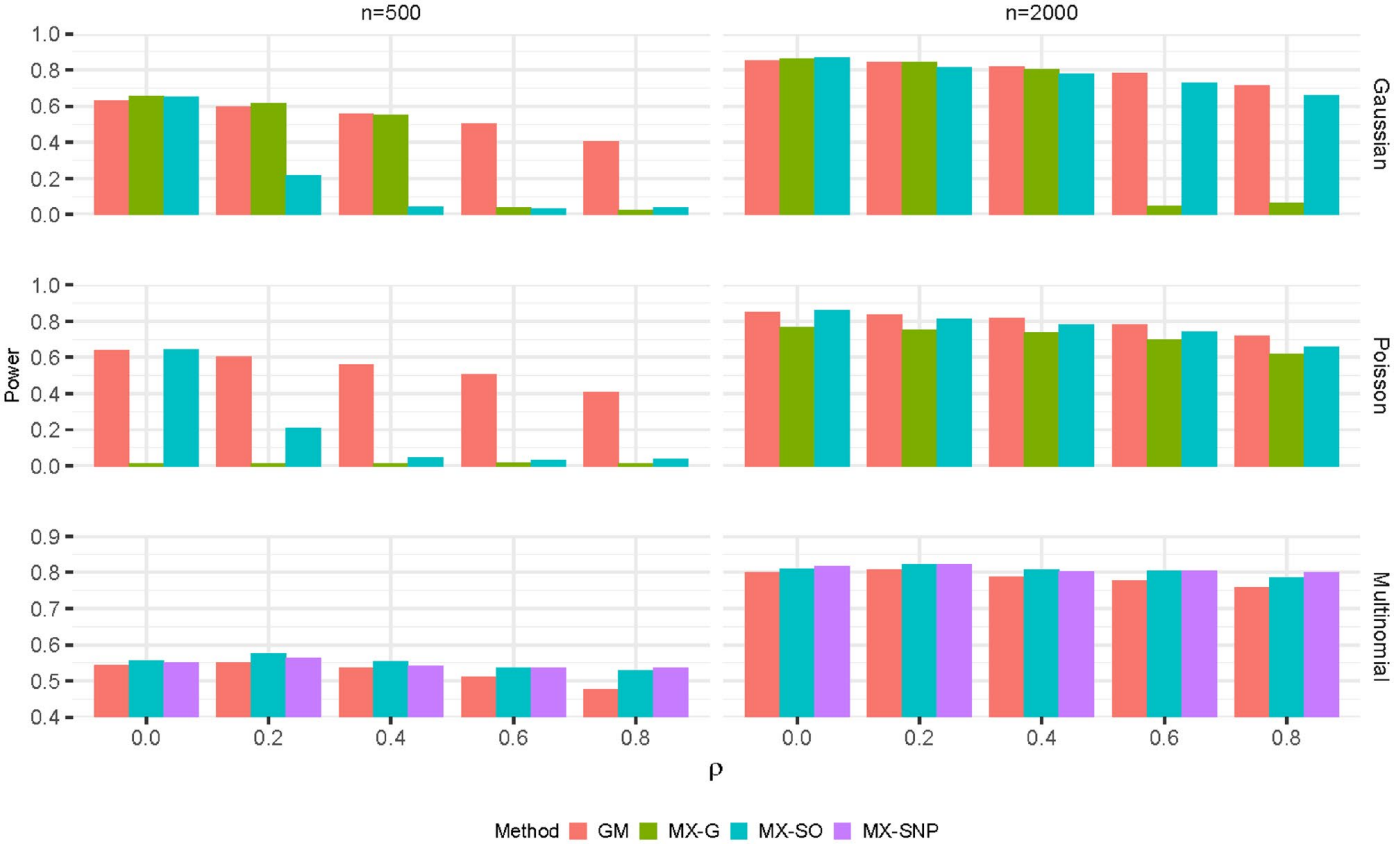
The power as the correlations among explanatory variables increase (FDR = 0.1).

Figure 2 and Supplementary Fig. S6 present the power as the correlations among explanatory variables increase when the target FDR is set to 0.1 and 0.2, respectively. When the explanatory variables follow a multivariate normal distribution, as the correlations increase, the power for all methods decreases with the reduction in GM method being the minimum, regardless of the sample sizes. For example, the power for both MX-G and MX-SO can reduce from around 0.6 to almost zero when n = 500, whereas the power for GM only reduces from 0.6 to 0.4. When n = 2000, the power of MX-G can still drop substantially when the correlation is above 0.4 (i.e. power dropped from 0.8 to 0.05), whereas its reduction becomes much smaller for both GM and MX-SO methods (i.e. the power changes from 0.85 to 0.71 and 0.87 to 0.66 for GM and MX-SO, respectively). When the explanatory variables follow Poisson distribution, it follows the same trend observed in multivariate normal distribution. Note that the GM method can have a slightly inflated FDR for small sample sizes at a high correlation $\rho \ge 0.6$ for Poisson explanatory variables. Nevertheless, the impact of correlation on power is more significant on MX-based methods than GM method. For the SNP data, both MX-based and GM methods have similar level of performance as the correlation increases. However, as the FDR is severely inflated for both MX-based methods (i.e. MX-SO and MX-SNP), their power should be interpreted with caution.

### Real data application

To empirically compare MX-based and GM methods, we analyzed the data obtained from the Alzheimer's Disease Neuroimaging Initiative (ADNI)^36^ and Parkinson Progression Marker Initiative (PPMI)^37^ studies.

#### The analysis of ADNI data

The Alzheimer’s Disease Neuroimaging Initiative (ADNI) is a longitudinal, multi-site observational study that includes the participants with cognitively normal, early mild cognitive impairment, late mild cognitive impairment or AD from 57 sites in the USA and Canada^36^. The blood DNA samples were drawn from study participants, and they were processed using Illumina’s non-CLIA whole-genome sequencing. The study was approved by the institutional review boards of all included ADNI centers (see full list: http://adni.loni.usc.edu). Informed consent was obtained from all subjects. The ADNI study and this work were carried out in accordance with relevant guidelines and regulations.

We focused on detecting SNPs that are associated with the baseline AV45, which is a PET-imaging outcome associated with AD. For the data, we performed quality control. We excluded genetic variants that met any of the following criteria: 1) missing genotype rate of individuals > 0.1; 2) missing genotype rate of SNP > 0.1; 3) the p-value of Hardy–Weinberg Equilibrium test ≤ 10–8; and 4) minor allele frequency of SNP < 0.01. We imputed the remaining missing genotypes using the mode.

As we focused on baseline data, we removed individuals with missing phenotypes, and a total of 520 individuals remained in our analyses. The distribution of AV45 is shown in Supplementary Fig. S7. Since neither GM nor knockoff methods can handle genome-wide data, we included all SNPs from 30 genes that have potential relationships with AD based on existing literature. The details of these genes are listed in Supplementary Table S5. To further reduce the dimension of the input data, we removed genetic variants with LD > 0.8, and a total of 1236 SNPs were included in the final analyses. We considered a gene being associative if any of the SNPs located on the gene is claimed significant. Since both procedures involve randomness either due to the generation of knockoff variables or mirror variables, we repeated the analysis 100 times to explore the chance of each gene being claimed significant. We set the targeted FDR at 0.1, and any genes with less than 5% being selected among 100 replicates are deemed not associated. As the MX-G method requires the assumption that the distribution of the explanatory variables is known and follows Gaussian distribution, the SNP data clearly do not satisfy this assumption. Therefore, as in the simulation study, we did not consider the MX-G method when analyzing the SNP data.

**Figure 3:**
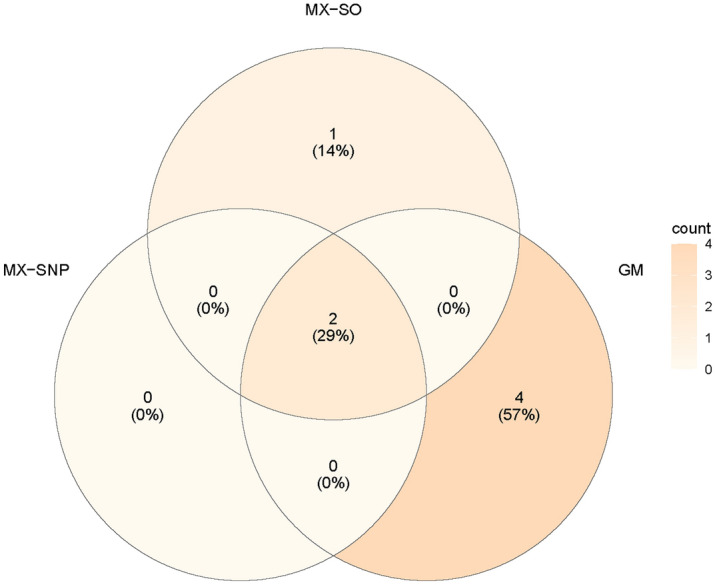
The consistency among AV45-associated genes selected by model-X knockoff models and Gaussian mirror.

The most highly selected genes about AV45 are shown in Table S5 and the comparisons of the selected genes among MX-based and GM methods are shown in Fig. 3. It can be seen in the results that MX-SNP and MX-SO methods are generally consistent. MX-SNP detected three genes that include *APOE* (100%), *ATF7* (20%) and *ADAM9* (5%), and both *APOE* (100%) and *ATF7* (17%) are also selected by MX-SO. 93.3% and 90% of the genes were never selected by the MX-SNP and MX-SO methods. GM method has detected 6 genes in total (Table S5), with *APOE* (99%), *ATF7* (59%) and *TOMM40* (35%) being detected more than 30%. About 80% of genes have never been selected by the GM method. Comparing GM with MX-based method, the most important genes tend to be consistently detected. For example, the well-known *APOE* has been selected by all three methods. Strong evidence suggests that APOE*ε4 carriers tend to have earlier and more abundant amyloid pathology in their brains. Individuals carrying APOE*ε4 are not only more likely to develop AD, but also may move forward the onset of AD^38^. The other gene selected by all the methods is ATF7, which has shown to be significantly associated with the risk for late onset AD. In addition, evidences have also suggested that there is an interaction effect between polymorphisms on ATF7 and APOE on the risk of AD^39^. However, there are some differences among the genes selected by the GM method and MX-based methods. For example, *TOMM40* has been rarely selected by the traditional knockoff methods, but it has a relatively high chance being selected by GM.

#### The analysis of PPMI data

The Parkinson Progression Marker Initiative (PPMI) is a comprehensive observational, international and multicenter study, which conducted longitudinally across 21 clinical sites using a standardized data collection protocol for clinical, imaging and biomarker assessment. Participants in the study include Parkinson's disease (PD), healthy controls, and carriers of the most common Parkinson's disease-related genetic mutations^40^. The study was approved by the steering committee. Informed consent was obtained from all subjects from the PPMI study and this work was carried out in accordance with relevant guidelines and regulations. All study data is publicly available through the PPMI website (http://www.ppmi-info.org/).

For PPMI, we focused on the T-tau of cerebrospinal fluid (CSF), which is a biomarkers of PD. We used the same quality control criteria as we used for the analysis of ADNI, and only focused on the baseline data. We removed individuals with missing phenotypes or missing genotypes, and a total of 838 individuals remained in our analyses. The distribution of T-tau assay for the 838 individuals is shown in Supplementary Fig. S8. We included all SNPs from 23 genes that have potential relationships with PD based on existing literature and removed genetic variants with LD > 0.8. 812 SNPs were included in the final analysis. The details are listed in Supplementary Table S6. Similar to the analysis of ADNI data, to reduce the impact of randomness caused by the generation of knockoff and mirror variables, we repeated the analysis 100 times. We set the targeted FDR at 0.1, and any genes with less than 5% being selected among 100 replicates are deemed not associated.

**Figure 4:**
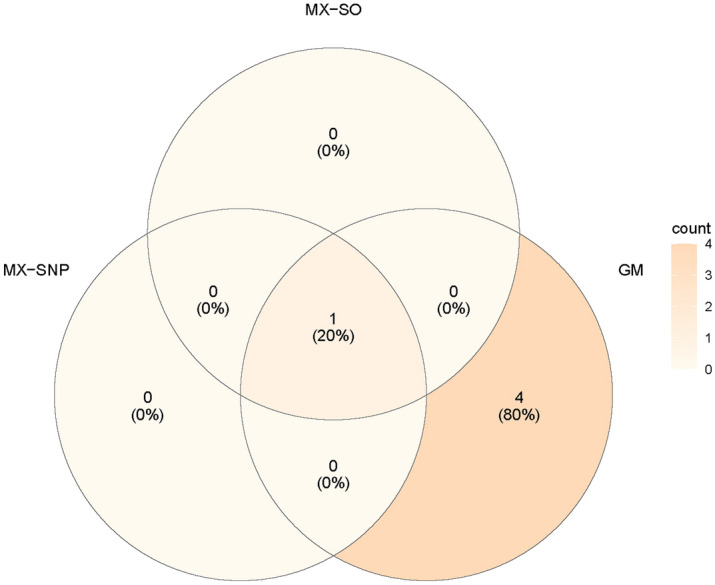
The consistency among PPMI-associated genes selected by model-X knockoff models and Gaussian mirror.

Table S6 presents the highly significant genes for T-tau and Fig. 4 summarizes the comparisons of the selected genes among MX-based and GM methods. MX-SNP and MX-SO methods behave similarly. The *KALRN* was selected with 81% and 52% by the MX-SNP and MX-SO methods, respectively. The rest of the 22 genes were never detected by both MX-SNP and MX-SO methods. A total of 3 genes was detected by the GM method by more than 10% of the times, including *ARSB* (57%), *KALRN* (15%) and *PCDHA9* (14%). About 78% of the genes were never detected by the GM method. The important gene *KALRN* was detected by all three methods. Existing studies have shown that *KALRN* plays a key role in the nervous system and is associated with various diseases such as stroke, schizophrenia and adult attention deficit-hyperactivity disorder^41^. *ARSB* that is shown to be associated with PD susceptibility by RNAi-mediated knockdown experiments^42^ is only detected by the GM method, not the MX-based methods.

## Discussion

In this paper, we conducted extensive simulation studies to systematically compare the performance of GM and MX-based methods, where the impacts of the distribution of explanatory variables, their relatedness and the signal-to-noise ratio were evaluated. We also compared the consistencies and discrepancies between them in detecting SNPs that are associated with AV45 and T-tau. Since GM and MX-based methods are recent advances in the field, our analyses have provided recommendations for their practical use.

For big biological data, the explanatory variables can come from various distributions. For example, the genomic data usually follows a multinomial distribution, and the transcriptomic data can be approximately by a Poisson distribution^43–45^. As the MX-based methods utilize the joint distribution of explanatory variables to construct valid knockoffs and GM requires the covariance among explanatory variables to be bounded, the distribution of the explanatory variables can apparently have an impact on their performance. We have found that all methods are inadequate in controlling FDR for the extremely heavy tailed Cauchy distribution. For other distributions, the GM method has well controlled FDR, and it usually has similar or slightly worse power than the best MX model, regardless of sample sizes. The MX with knockoff generated using the second-order approximates (i.e. MX-SO) generally performs better than the corresponding MX with knockoff generated using the Gaussian distribution. However, the performance of MX-SO can depend on the sample size, and its performance can be significantly worse than GM method for small sample sizes, partially due to unreliable mean and covariance estimate. In addition, we have noticed that although MX-SNP is particularly designed for SNP data, its performance is very similar to MX-SO, suggesting MX-SO has a reasonably robust performance across a range of distributions. Therefore, regardless of distributions, for independent explanatory variables, we believe that the MX-SO method is powerful when the sample size is relatively large or the explanatory variables are known to follow Gaussian distributions. However, when the distribution is unknown and the sample size is limited, the GM method is a robust and powerful alternative.

For a particular application, the amount of noise can be quite different. For example, the amount of noise variables in the candidate gene-based studies can be substantially smaller than those in the genome-wide studies^46,47^. To evaluate its impact, we simulated the data with signal-to-noise ratios. We found that the amount of noise has little impact on the FDR and all methods controlled the FDR at the target level, although MX-G is conservative when explanatory variables are not from Gaussian distributions. As expected, the power for all methods decreases as the amount of noise increases, and the reduction in power is much more apparent in MX-G. GM, MX-SO and MX-SNP have the similar level of performance, and the reduction in power as the amount of noise increases is also similar among them.

Of particular note, we have found that the performance of MX-SO can depend on how the covariance matrix is estimated. Figure S9 shows the FDR and power when the covariance is estimated by the traditional maximum-likelihood estimator and the James–Stein-type shrinkage estimator^48^ for a target FDR rate of 0.1. Given a total of 1200 independent explanatory variables, with covariance estimated using the traditional maximum likelihood estimators, the power of MX increases as the sample sizes increase until the sample sizes are close to the number of explanatory variables. It then drops substantially when the sample sizes are close to the number of explanatory variables, and then gradually increases as the sample sizes further increase. This trend holds regardless of the distribution of explanatory variables. When the covariance is estimated using the James–Stein-type shrinkage estimator, the power of MX is not changed significantly, but increases steadily with the increase of sample size. This is an interesting phenomenon that can have a major impact on the performance of MX-SO method. It is well-known that for high-dimensional data (i.e. *p* > *n*), the maximum likelihood estimation (MLE) for the covariance is a poor estimate. For example, when *p* > *n*, the eigenvalue of covariance estimated from MLE can be far away from the true covariance. While the shrinkage estimator of covariance matrix is not necessarily consistent, it is well-conditioned and can improve performance for lots of tasks. Therefore, under the settings that the sample size is smaller than the number of variables, we recommend to use robust methods for covariance estimation so that the power for MX-SO can be optimized.

The relatedness among explanatory variables is a quite common phenomenon in big biological data. For example, in the analysis of human neuroblastoma, Longo L et al*.* found *APOC1P1*, *TOMM40* and *PVRL2* are highly co-expressed^49^. Similarly, it is well known that some SNPs can have high LD (e.g. rs1316356 and rs9877502^50^). However, the MX-based methods assume independence among explanatory variables and the GM method only allows weak correlations, and thus the assumptions employed by both MX-based and GM methods can be violated. To assess its impact, we compared MX-based and GM methods given different levels of relatedness among explanatory variables. The GM method has well controlled FDR regardless of the distribution of explanatory variables when sample size is large, but it tends to be inflated for non-Gaussian variables when the sample size is small and the correlation is high (i.e. > 0.6). On contrary, the MX-based methods either have severely inflated FDR or become over conservative as the correlation increases, and the deviation from the desired FDR target rate is much larger when the sample size is small. As MX-based methods are not capable of appropriately controlling the FDR, we recommend using the GM method when there are moderate correlations among explanatory variables. We also suggest to remove highly correlated variables (e.g. pairwise correlation > 0.6) before the analysis for the GM method, especially when the sample size is relatively small.

We have applied both MX-based and GM methods to detect SNPs that are associated with AV45 and T-tau. For AV45, we found that MX-SO and MX-SNP methods were highly consistent. *APOE*, *ATF7* were selected with high probability by these two methods. Comparing the results obtained from MX and GM methods, although the most important genes have been selected by both methods, there is a larger difference between MX-based and GM methods. For example, *TOMM40* was selected with a high probability by GM, but it has never been detected by the MX-based methods. As shown in Fig. 3, there is only 29% of consistency between the MX-based and GM methods. 57% of the genes that are selected by GM have never been selected by the MX-based methods. For T-tau, the well-known PD-related gene *KALRN* is selected by all three methods. While *KALRN* is the only gene selected by MX-based methods, GM has further detected an additional 2 genes (*ARSB* and *PCDHA9*). The consistency between GM and MX methods is considered low, as 80% of the genes that are selected by GM have never been selected by the MX-based methods (Fig. 4). Since we used LD < 0.8 to filter out highly correlated SNPs, the results from MX-based methods should be interpreted with caution. This is mainly because MX-based methods could have inappropriate level of FDR for SNP data given our current sample size, and further studies are needed to fully examine the selected SNPs.

We evaluated the computational time for each method, including the parallel computing with the number of cores set to be 1 and 4, respectively. The average running time for each method is calculated based on 100 replicates and the results are shown in Supplementary Table S7. In general, the computational time for MX-SO and GM methods are similar for continuous variables (i.e. Gaussian and Poisson), but MX-SO is substantially faster than the GM method for categorical variables. In contrast, the MX-SNP and MX-G is always much faster than GM method. Not surprisingly, with parallel computing implemented, the computational time has decreased for both methods, and the amount of reduction in computational time is similar for continuous variables. Despite that the computational time for GM reduces much faster as compared to MX-based methods for multinomial variables, it is still substantially slower than MX-based methods. This is mainly because the GM method performs a large number of calculations in order to find the scalar $c$, making the GM method computationally expensive. While the MX-based methods do not require to search the optimum value of $c$, the MX-SO requires the estimation of a covariance matrix, leading to a high computational cost. Unlike MX-SO, MX-G method does not need to rely on the estimation of the covariance matrix and MX-SNP derives its knockoffs based on fastphase, which is phasing and imputation tools used to assist Hidden Markov Model for the knockoff generation^51^. Therefore, both MX-G and MX-SNP are much more computationally efficient.

There are some limitations of this research. While we mainly focused on the comparisons between MX and GM methods that can be used for the analysis of big biological data, other innovative FDR control methods (e.g. data-splitting and multiple data-splitting^52^) are also worth investigated. In addition, we have primarily focused on linear models, and other models (e.g. generalized linear model and survival analysis) can also be of interest in practice. These can be a future direction of our research.

The key characteristics of MX-based and GM methods are presented in Table 4. In summary, GM method is a robust and powerful method, especially for data with small sample size, unknown distribution of explanatory variables and high correlation among variables. It tends to be more computationally expensive as compared to MX-G and MX-SNP. While MX-G tends to be computationally efficient, we do not recommend using the MX-G, except when the explanatory variables are known to follow Gaussian distribution. When the sample size is large and the correlation among explanatory variables is relatively small, both GM and MX-SO can be used and their computational cost are similar except for SNP data, where MX-SO is much faster. Although MX-SO tends to have slightly higher power than GM under these conditions, the validity of covariance estimation needs to be considered.

**Table 4:** Details of the use of MX-based and GM methods.

Method	MX-G	MX-SO	MX-SNP	GM
Distribution of explanatory variables	Gaussian distribution	Various distributions for which means and variances can be estimated	SNP data	Various distributions
Correlation between explanatory variables	Low correlation	Low correlation	Medium/low correlation	Medium/low correlation
Computational time	Fast	Slow	Fast	Slow

## Supplementary Information

Supplementary Information.

## Data Availability

The data analyzed in this study is subject to the following licenses/restrictions: The datasets can be found at http://adni.loni.ucla.edu/data-samples/access-data for ADNI and http://www.ppmi-info.org/ for PPMI, and they can be requested from ADNI and PPMI studies. Requests to access this dataset should be directed to ADNI: http://adni.loni.ucla.edu/, PPMI: http://www.ppmi-info.org/.
