# Supplementary material for: The systematic comparison between Gaussian mirror and Model-X knockoff models
Source: Sci Rep. 2023 Apr 4;13:5478. doi: 10.1038/s41598-023-32605-5 (PMC10073103; doi:10.1038/s41598-023-32605-5)
Supplement: Supplementary file 1 — Supplementary Information. [file 41598_2023_32605_MOESM1_ESM.docx]

**Supplementary tables**

Table S1. The false discovery rate when the explanatory variables follow different distributions (n=2,000).

| Distributions | Model X-knockoff | | | GM |
| --- | --- | --- | --- | --- |
|  | Gaussian | Second-order | SNPs |  |
| Target FDR = 0.2 | | | | |
| Gaussian | 0.207 | 0.203 | —— | 0.210 |
| Poisson | 0.145 | 0.216 | —— | 0.203 |
| Uniform | 0.213 | 0.219 | —— | 0.208 |
| Cauchy | 0.681 | 0.214 | —— | 0.077 |
| Multinomial | —— | 0.204 | 0.220 | 0.191 |
| Target FDR = 0.1 | | | | |
| Gaussian | 0.105 | 0.112 | —— | 0.109 |
| Poisson | 0.042 | 0.120 | —— | 0.111 |
| Uniform | 0.113 | 0.120 | —— | 0.107 |
| Cauchy | 0.599 | 0.148 | —— | 0.053 |
| Multinomial | —— | 0.108 | 0.122 | 0.111 |

Table S2. The power when the explanatory variables follow different distributions (Target FDR=0.2).

| Distributions | Model-X knockoff | | | GM |
| --- | --- | --- | --- | --- |
|  | Gaussian | Second-order | SNPs |  |
| n=500 | | | | |
| Gaussian | 0.688 | 0.680 | —— | 0.663 |
| Poisson | 0.019 | 0.674 | —— | 0.665 |
| Uniform | 0.020 | 0.374 | —— | 0.664 |
| Multinomial | —— | 0.589 | 0.589 | 0.572 |
| n=2000 | | | | |
| Gaussian | 0.876 | 0.880 | —— | 0.866 |
| Poisson | 0.796 | 0.876 | —— | 0.867 |
| Uniform | 0.881 | 0.877 | —— | 0.868 |
| Multinomial | —— | 0.830 | 0.836 | 0.814 |

Table S3. The false discovery rate as the number of noise variables increase.

| Sample size | The number of variables | Model-X knockoff | | | | | | GM | | |
| --- | --- | --- | --- | --- | --- | --- | --- | --- | --- | --- |
|  |  | Gaussian | | Second-order | | | SNPs |  |  |  |
|  |  | N(0,1) | Poi(3) | N(0,1) | Poi(3) | SNPs | SNPs | N(0,1) | Poi(3) | SNPs |
| N=500 | Target FDR = 0.2 | | | | | | | | | |
|  | 200 | 0.195 | 0.128 | 0.184 | 0.190 | 0.195 | 0.181 | 0.170 | 0.174 | 0.181 |
|  | 400 | 0.197 | 0.002 | 0.195 | 0.198 | 0.216 | 0.202 | 0.192 | 0.194 | 0.199 |
|  | 800 | 0.202 | 0.000 | 0.205 | 0.206 | 0.209 | 0.203 | 0.187 | 0.212 | 0.194 |
|  | 1200 | 0.203 | 0.000 | 0.215 | 0.212 | 0.224 | 0.180 | 0.175 | 0.203 | 0.191 |
|  | 2000 | 0.206 | 0.000 | 0.227 | 0.212 | 0.217 | 0.212 | 0.191 | 0.160 | 0.166 |
|  | 3000 | 0.215 | 0.000 | 0.212 | 0.230 | 0.243 | 0.262 | 0.178 | 0.158 | 0.148 |
|  | 10000 | 0.195 | 0.000 | 0.202 | 0.193 | 0.258 | 0.239 | 0.115 | 0.082 | 0.113 |
|  | Target FDR=0.1 | | | | | | | | | |
|  | 200 | 0.108 | 0.048 | 0.099 | 0.100 | 0.106 | 0.095 | 0.100 | 0.099 | 0.108 |
|  | 400 | 0.105 | 0.000 | 0.103 | 0.110 | 0.131 | 0.111 | 0.106 | 0.110 | 0.112 |
|  | 800 | 0.107 | 0.000 | 0.106 | 0.102 | 0.111 | 0.107 | 0.097 | 0.123 | 0.111 |
|  | 1200 | 0.109 | 0.000 | 0.114 | 0.111 | 0.120 | 0.088 | 0.099 | 0.115 | 0.119 |
|  | 2000 | 0.104 | 0.000 | 0.120 | 0.119 | 0.114 | 0.106 | 0.117 | 0.088 | 0.108 |
|  | 3000 | 0.112 | 0.000 | 0.101 | 0.120 | 0.158 | 0.164 | 0.110 | 0.094 | 0.089 |
|  | 10000 | 0.117 | 0.000 | 0.117 | 0.153 | 0.152 | 0.166 | 0.054 | 0.064 | 0.052 |
| N=2000 | Target FDR=0.2 | | | | | | | | | |
|  | 200 | 0.216 | 0.171 | 0.202 | 0.196 | 0.193 | 0.206 | 0.175 | 0.182 | 0.173 |
|  | 400 | 0.211 | 0.189 | 0.200 | 0.201 | 0.187 | 0.205 | 0.196 | 0.191 | 0.190 |
|  | 800 | 0.209 | 0.205 | 0.216 | 0.215 | 0.208 | 0.209 | 0.202 | 0.199 | 0.206 |
|  | 1200 | 0.207 | 0.145 | 0.203 | 0.216 | 0.204 | 0.220 | 0.210 | 0.203 | 0.191 |
|  | 2000 | 0.206 | 0.000 | 0.200 | 0.209 | 0.222 | 0.209 | 0.201 | 0.195 | 0.198 |
|  | 3000 | 0.215 | 0.000 | 0.214 | 0.206 | 0.250 | 0.235 | 0.202 | 0.209 | 0.166 |
|  | 10000 | 0.219 | 0.000 | 0.215 | 0.224 | 0.227 | 0.263 | 0.188 | 0.190 | 0.146 |
|  | Target FDR=0.1 | | | | | | | | | |
|  | 200 | 0.106 | 0.084 | 0.104 | 0.099 | 0.106 | 0.104 | 0.103 | 0.104 | 0.103 |
|  | 400 | 0.108 | 0.090 | 0.100 | 0.105 | 0.101 | 0.101 | 0.104 | 0.098 | 0.114 |
|  | 800 | 0.112 | 0.093 | 0.112 | 0.113 | 0.111 | 0.106 | 0.108 | 0.105 | 0.117 |
|  | 1200 | 0.105 | 0.042 | 0.112 | 0.120 | 0.108 | 0.122 | 0.109 | 0.111 | 0.111 |
|  | 2000 | 0.109 | 0.000 | 0.100 | 0.108 | 0.121 | 0.114 | 0.106 | 0.096 | 0.105 |
|  | 3000 | 0.113 | 0.000 | 0.105 | 0.107 | 0.154 | 0.154 | 0.108 | 0.117 | 0.090 |
|  | 10000 | 0.101 | 0.000 | 0.111 | 0.119 | 0.161 | 0.168 | 0.104 | 0.116 | 0.083 |

Table S4. The false discovery rate as correlations among explanatory variables increase (n=2,000).

| The correlation of variables | Model-X knockoff | | | | | | GM | | |
| --- | --- | --- | --- | --- | --- | --- | --- | --- | --- |
|  | Gaussian | | Second-order | | | SNPs |  |  |  |
|  | N(0,1) | Poi(3) | N(0,1) | Poi(3) | SNPs | SNPs | N(0,1) | Poi(3) | SNPs |
| Target FDR = 0.2 | | | | | | | | | |
| 0 | 0.207 | 0.145 | 0.203 | 0.216 | 0.204 | 0.220 | 0.210 | 0.203 | 0.191 |
| 0.2 | 0.202 | 0.157 | 0.190 | 0.193 | 0.213 | 0.222 | 0.202 | 0.194 | 0.193 |
| 0.4 | 0.208 | 0.181 | 0.178 | 0.186 | 0.250 | 0.228 | 0.210 | 0.198 | 0.199 |
| 0.6 | 0.003 | 0.194 | 0.182 | 0.181 | 0.250 | 0.252 | 0.218 | 0.203 | 0.204 |
| 0.8 | 0.012 | 0.219 | 0.169 | 0.166 | 0.250 | 0.302 | 0.223 | 0.209 | 0.200 |
| Target FDR = 0.1 | | | | | | | | | |
| 0 | 0.105 | 0.042 | 0.112 | 0.120 | 0.108 | 0.122 | 0.109 | 0.111 | 0.111 |
| 0.2 | 0.107 | 0.048 | 0.098 | 0.095 | 0.108 | 0.112 | 0.113 | 0.112 | 0.098 |
| 0.4 | 0.098 | 0.068 | 0.087 | 0.089 | 0.144 | 0.122 | 0.113 | 0.112 | 0.109 |
| 0.6 | 0.004 | 0.070 | 0.081 | 0.084 | 0.152 | 0.161 | 0.114 | 0.111 | 0.117 |
| 0.8 | 0.006 | 0.082 | 0.076 | 0.068 | 0.158 | 0.211 | 0.116 | 0.115 | 0.125 |

Table S5. Genes used in the analysis of ADNI dataset.

| Gene | Method | | |
| --- | --- | --- | --- |
|  | MX-SNP | MX-SO | Gaussian Mirror |
| *APOE* | 1 | 1 | 0.99 |
| *ATF7* | 0.20 | 0.17 | 0.59 |
| *ADAM9* | 0 | 0.05 | 0 |
| *TOMM40* | 0 | 0 | 0.35 |
| *RELN* | 0 | 0 | 0.15 |
| *SLC11A2* | 0 | 0 | 0.13 |
| *NGF* | 0 | 0 | 0.05 |
| *APOC1* | 0 | 0 | 0 |
| *APOC1P1* | 0 | 0 | 0 |
| *APOC2* | 0 | 0 | 0 |
| *APOC3* | 0 | 0 | 0 |
| *APOC4* | 0 | 0 | 0 |
| *ATF7IP* | 0 | 0 | 0 |
| *CAV1* | 0 | 0 | 0 |
| *DFNB31* | 0 | 0 | 0 |
| *FGF1* | 0 | 0 | 0 |
| *FGF10* | 0 | 0 | 0 |
| *HBG2* | 0 | 0 | 0 |
| *HNRNPC* | 0 | 0 | 0 |
| *HSD11B1* | 0 | 0 | 0 |
| *HSPA1A* | 0 | 0 | 0 |
| *HSPA5* | 0 | 0 | 0 |
| *KLF5* | 0 | 0 | 0 |
| *MICA* | 0 | 0 | 0 |
| *MTHFD1* | 0 | 0 | 0 |
| *NAT1* | 0 | 0 | 0 |
| *POMT1* | 0 | 0 | 0 |
| *PON3* | 0 | 0 | 0 |
| *PPP2R2B-IT1* | 0 | 0 | 0 |
| *TREM2* | 0 | 0 | 0 |

Table S6. Genes used in the analysis of PPMI dataset.

| Gene | Method | | |
| --- | --- | --- | --- |
|  | MX-SNP | MX-SO | Gaussian Mirror |
| *KALRN* | 0.81 | 0.52 | 0.15 |
| *ARSB* | 0 | 0 | 0.57 |
| *PCDHA9* | 0 | 0 | 0.14 |
| *PZP* | 0 | 0 | 0.09 |
| *CD36* | 0 | 0 | 0.05 |
| *ANKRD30A* | 0 | 0 | 0 |
| *CALML4* | 0 | 0 | 0 |
| *CAPS2* | 0 | 0 | 0 |
| *COL6A5* | 0 | 0 | 0 |
| *DIS3* | 0 | 0 | 0 |
| *FAM71A* | 0 | 0 | 0 |
| *GH2* | 0 | 0 | 0 |
| *KCNK16* | 0 | 0 | 0 |
| *MNS1* | 0 | 0 | 0 |
| *OR7G3* | 0 | 0 | 0 |
| *PTPRH* | 0 | 0 | 0 |
| *SSPO* | 0 | 0 | 0 |
| *SVOPL* | 0 | 0 | 0 |
| *TCHHL1* | 0 | 0 | 0 |
| *TMEM134* | 0 | 0 | 0 |
| *UHRF1BP1L* | 0 | 0 | 0 |
| *VPS13C* | 0 | 0 | 0 |
| *ZNF543* | 0 | 0 | 0 |

Table S7. Computational time of different methods (unit: s).

| The number of cores | Distributions | Model-X knockoff | | | GM |
| --- | --- | --- | --- | --- | --- |
|  |  | Gaussian | Second-order | SNPs |  |
| 1 | Gaussian | 19.5 | 166.5 | —— | 167.9 |
|  | Poisson | 14.5 | 130.8 | —— | 143.2 |
|  | Multinomial | —— | 23.2 | 6.5 | 151.6 |
| 4 | Gaussian | 13.8 | 98.7 | —— | 95.3 |
|  | Poisson | 11.2 | 77.9 | —— | 70.6 |
|  | Multinomial | —— | 20.2 | 5.1 | 87.3 |

**Supplementary figures**

**
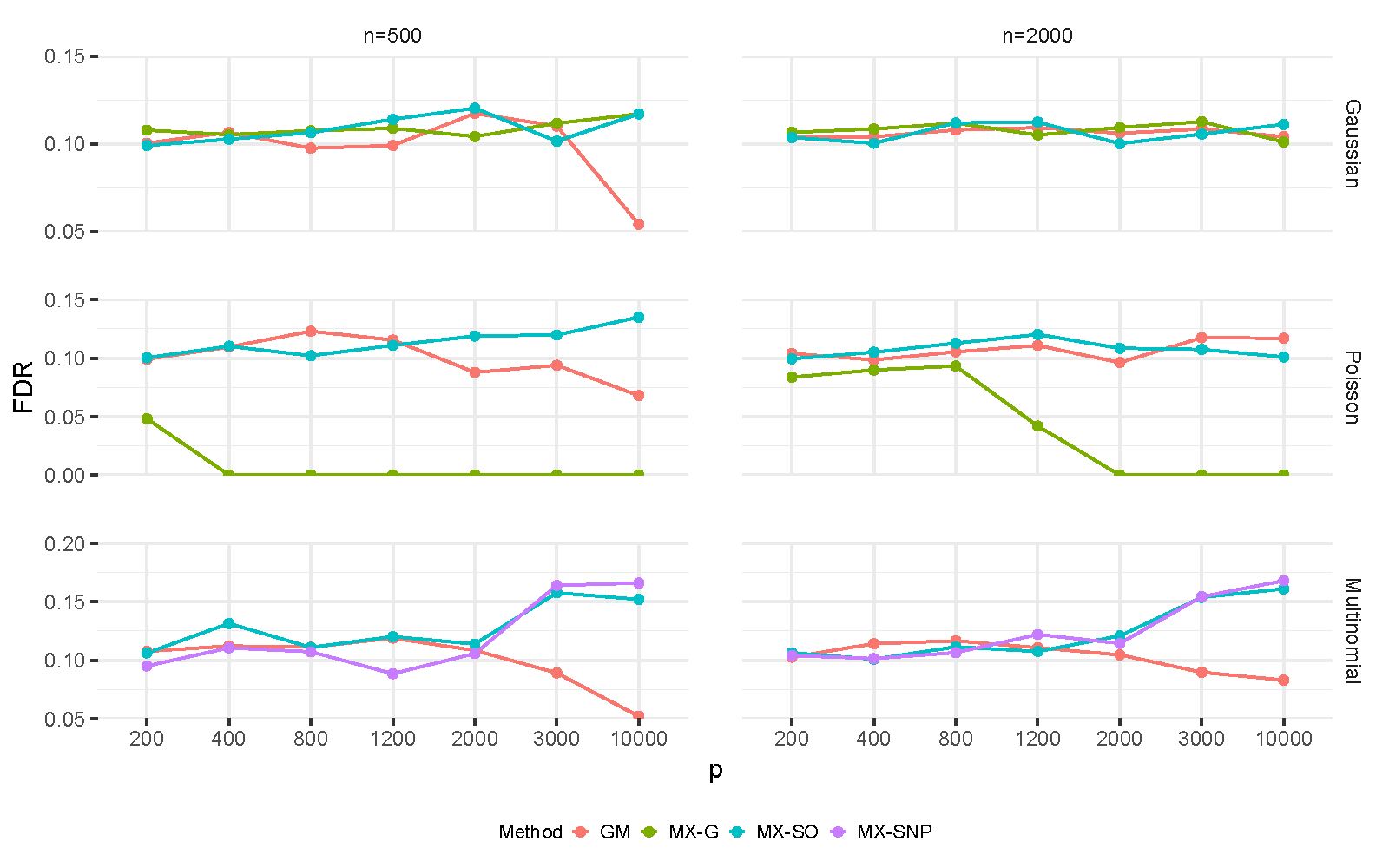
**

Figure S1. The false discovery rate as the number of noise variables increases (FDR=0.1)

**
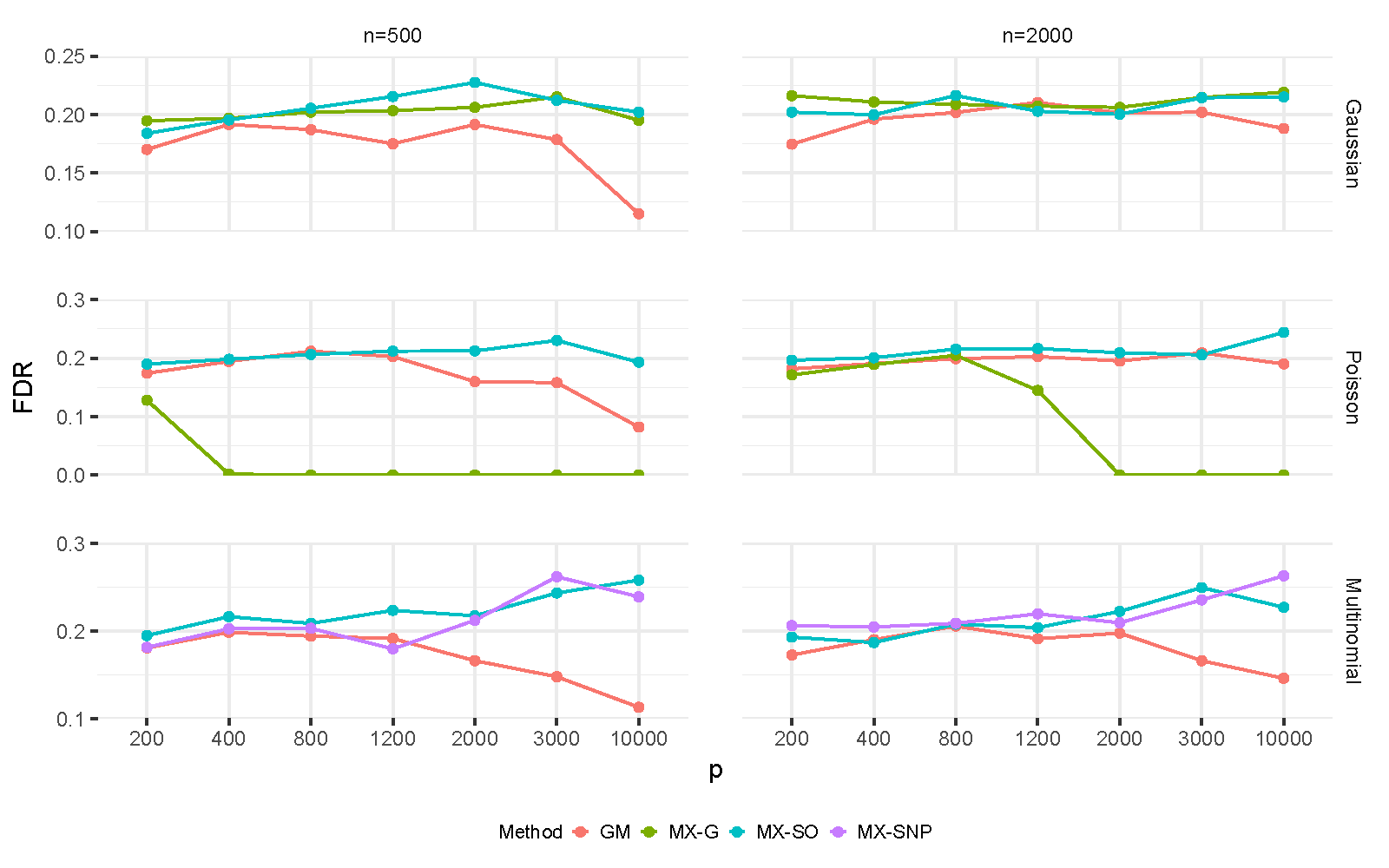
**

Figure S2. The false discovery rate as the number of noise variables increases (FDR=0.2).

**
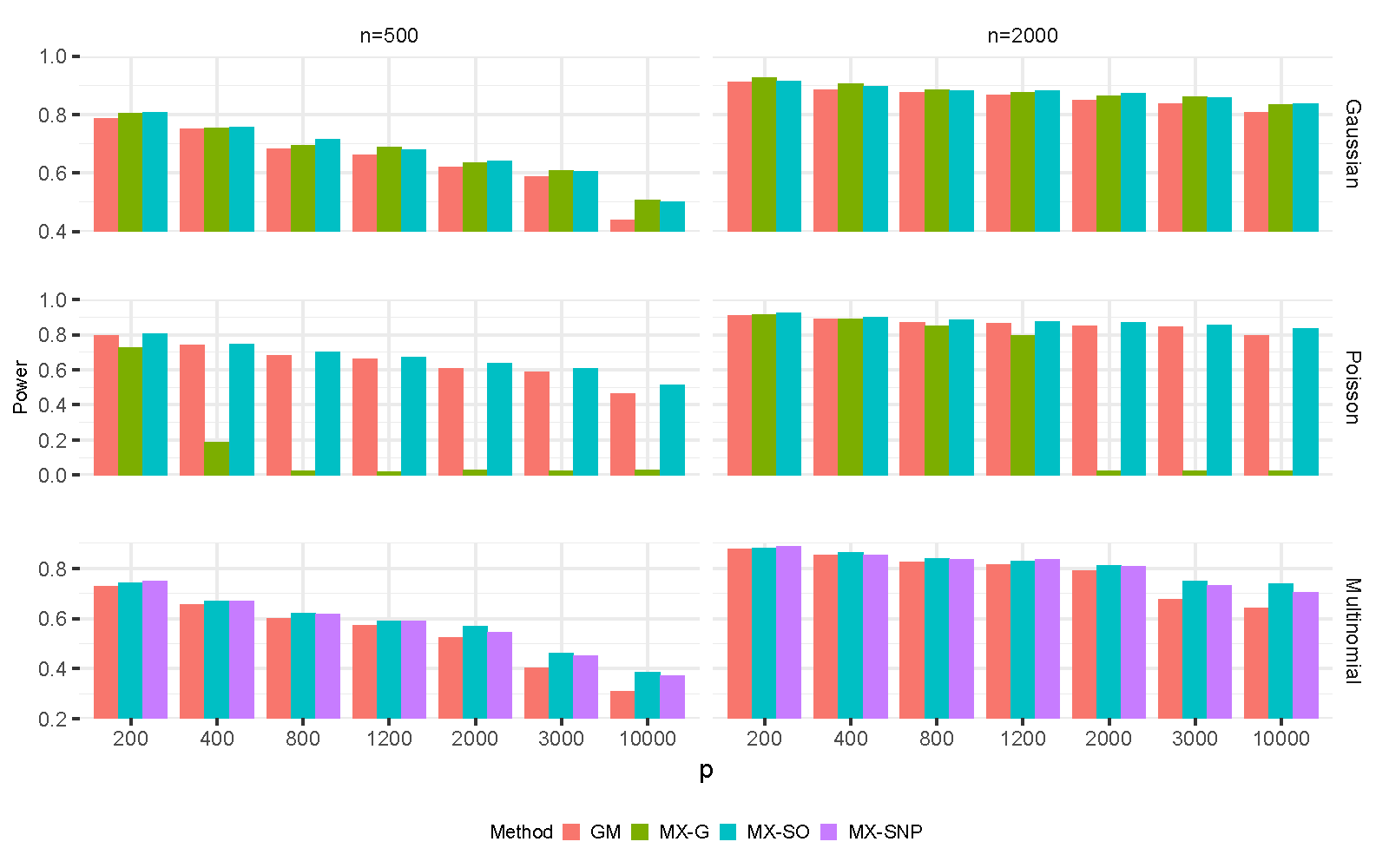
**

Figure S3. The power as the number of noise variables increases (FDR=0.2).


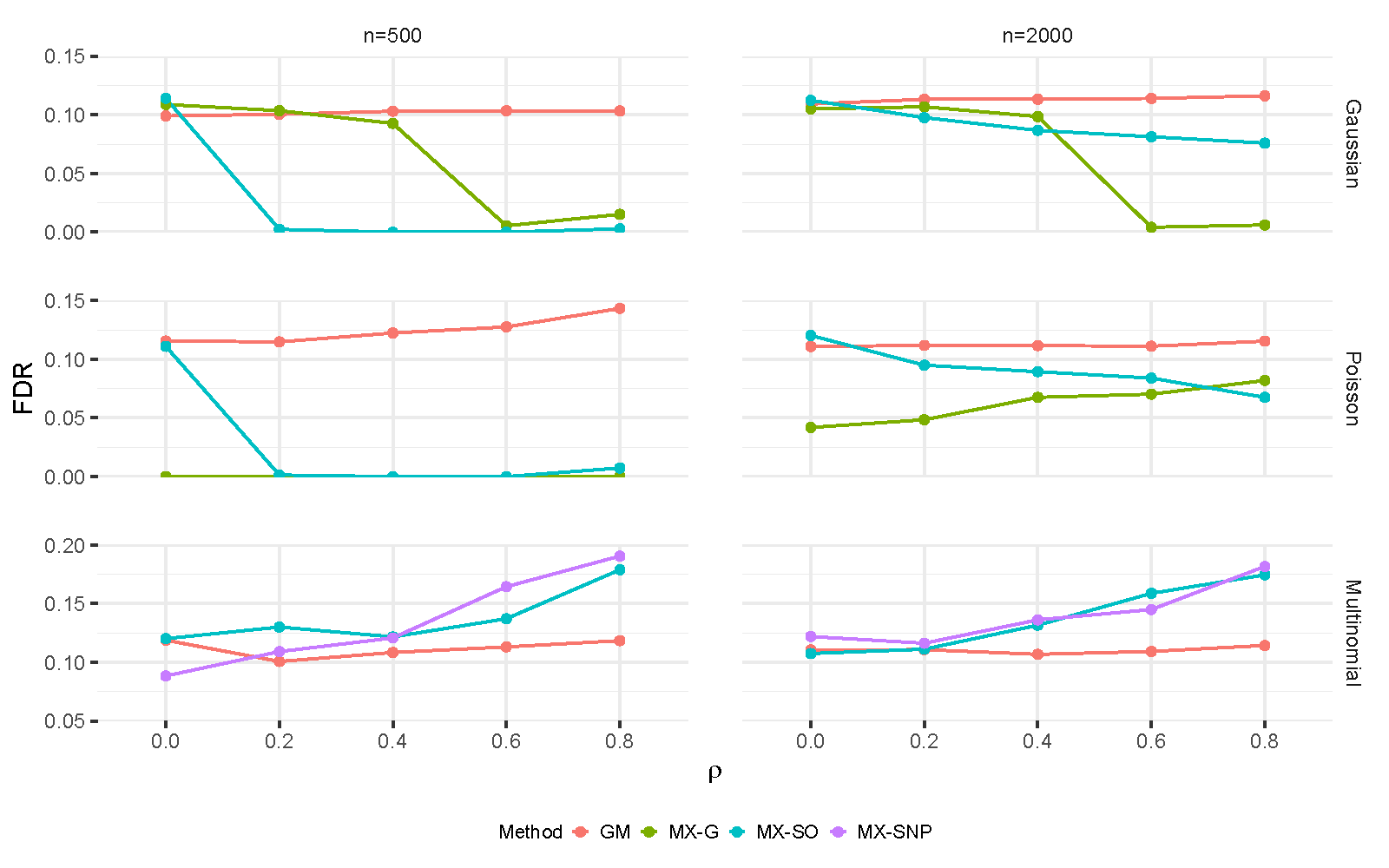


Figure S4. The false discovery rate as correlations among explanatory variables increase (FDR=0.1).


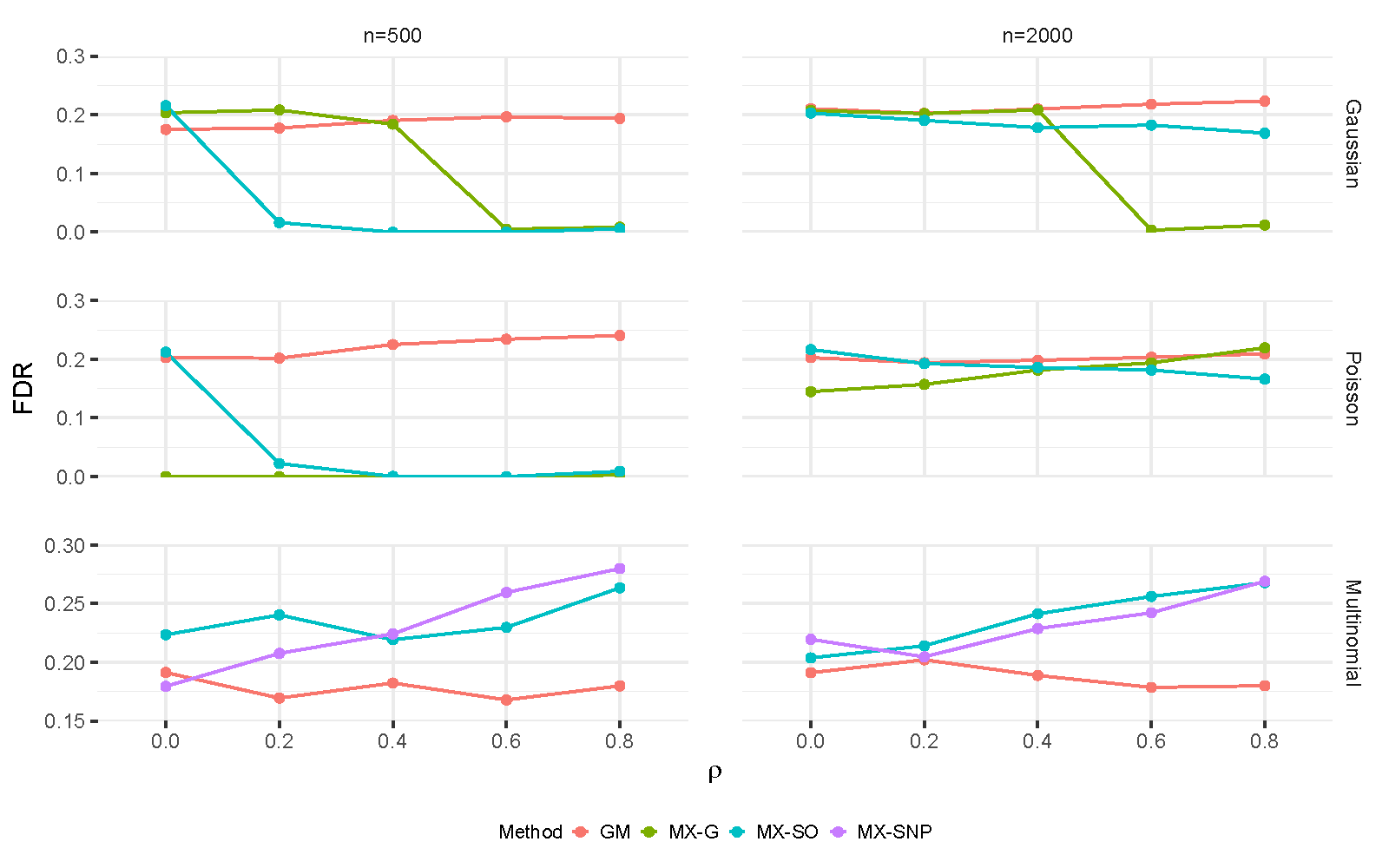


Figure S5. The false discovery rate as correlations among explanatory variables increase (FDR=0.2).


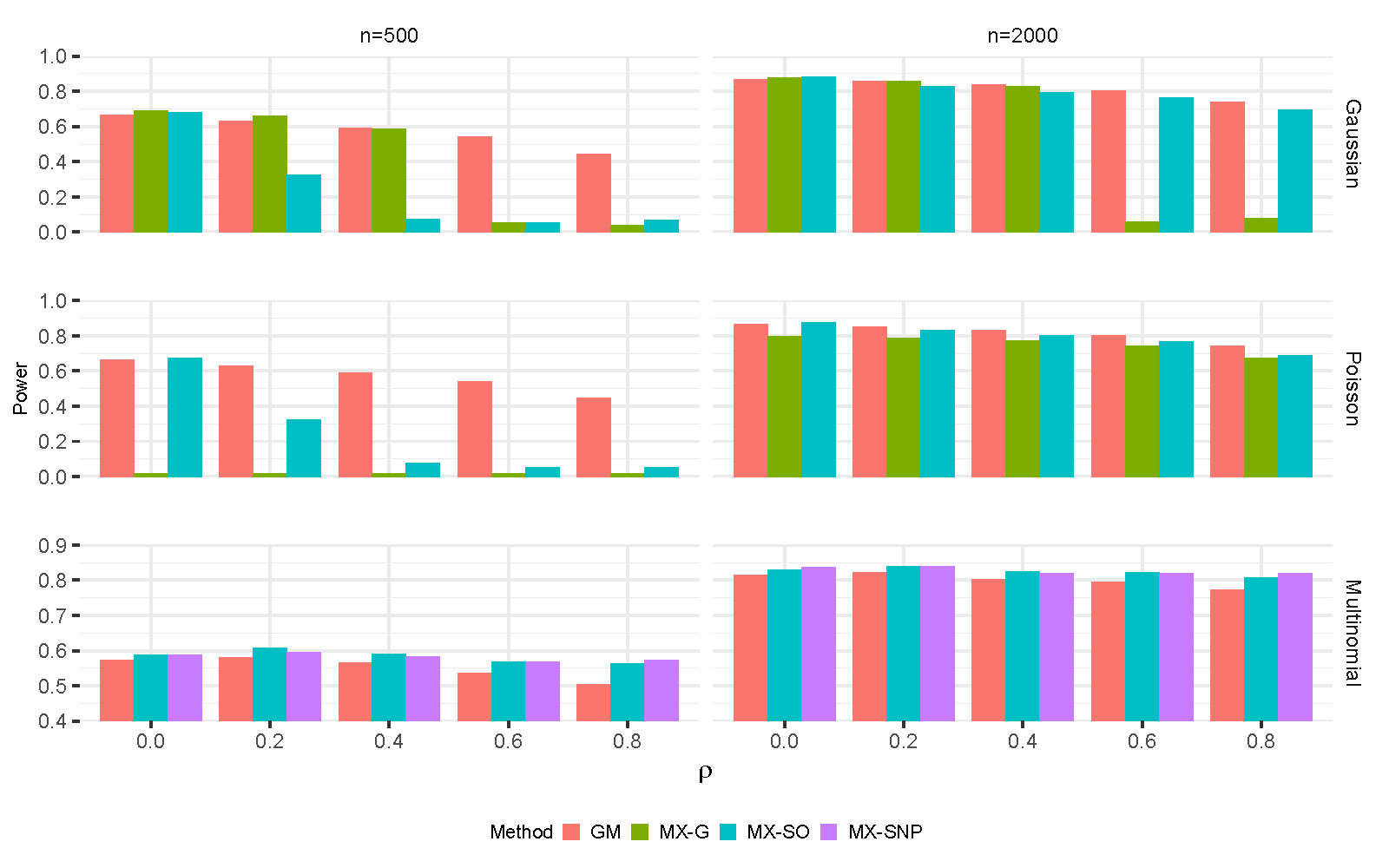


Figure S6. The power as the correlations among explanatory variables increase (FDR=0.2).


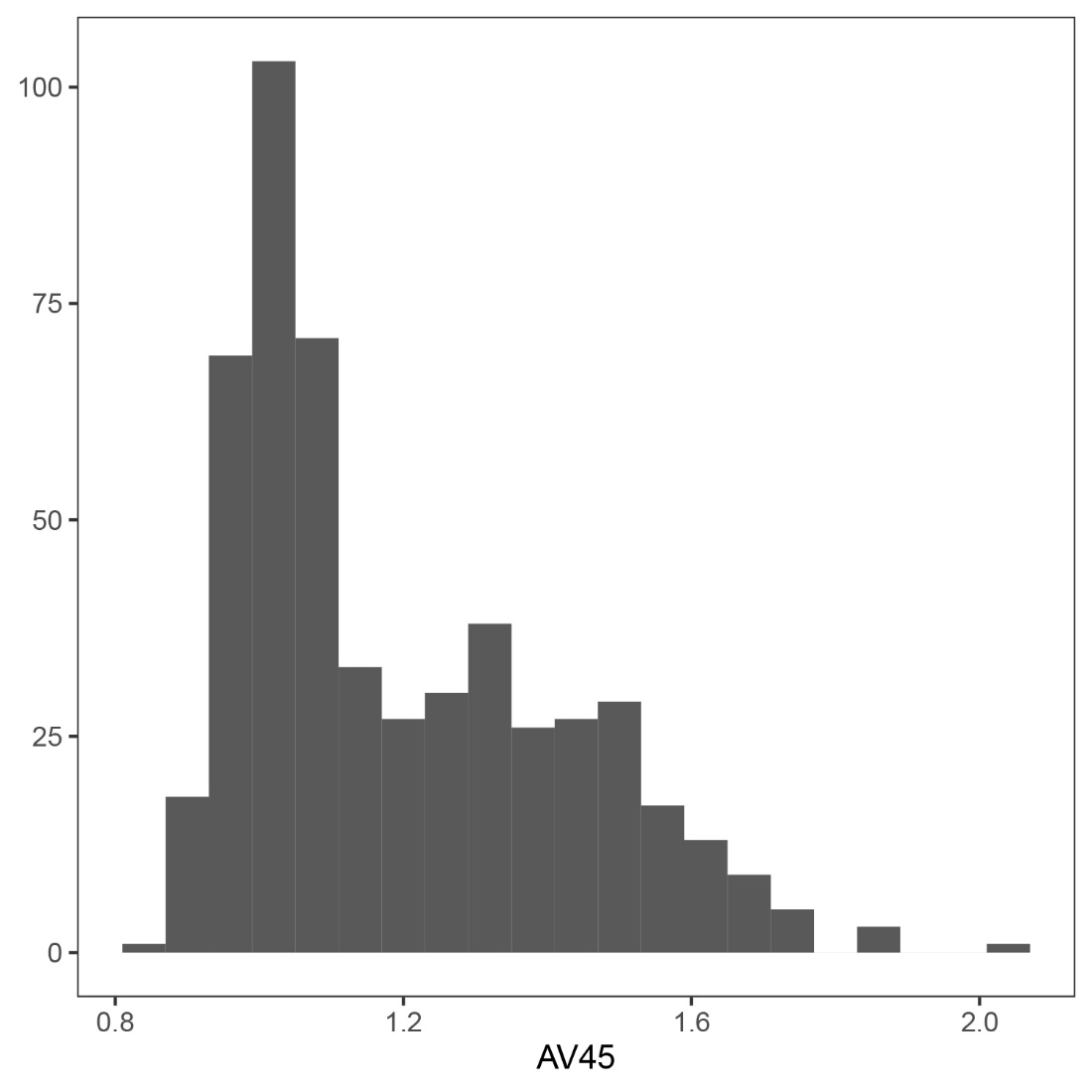


Figure S7. The distributions of AV45.


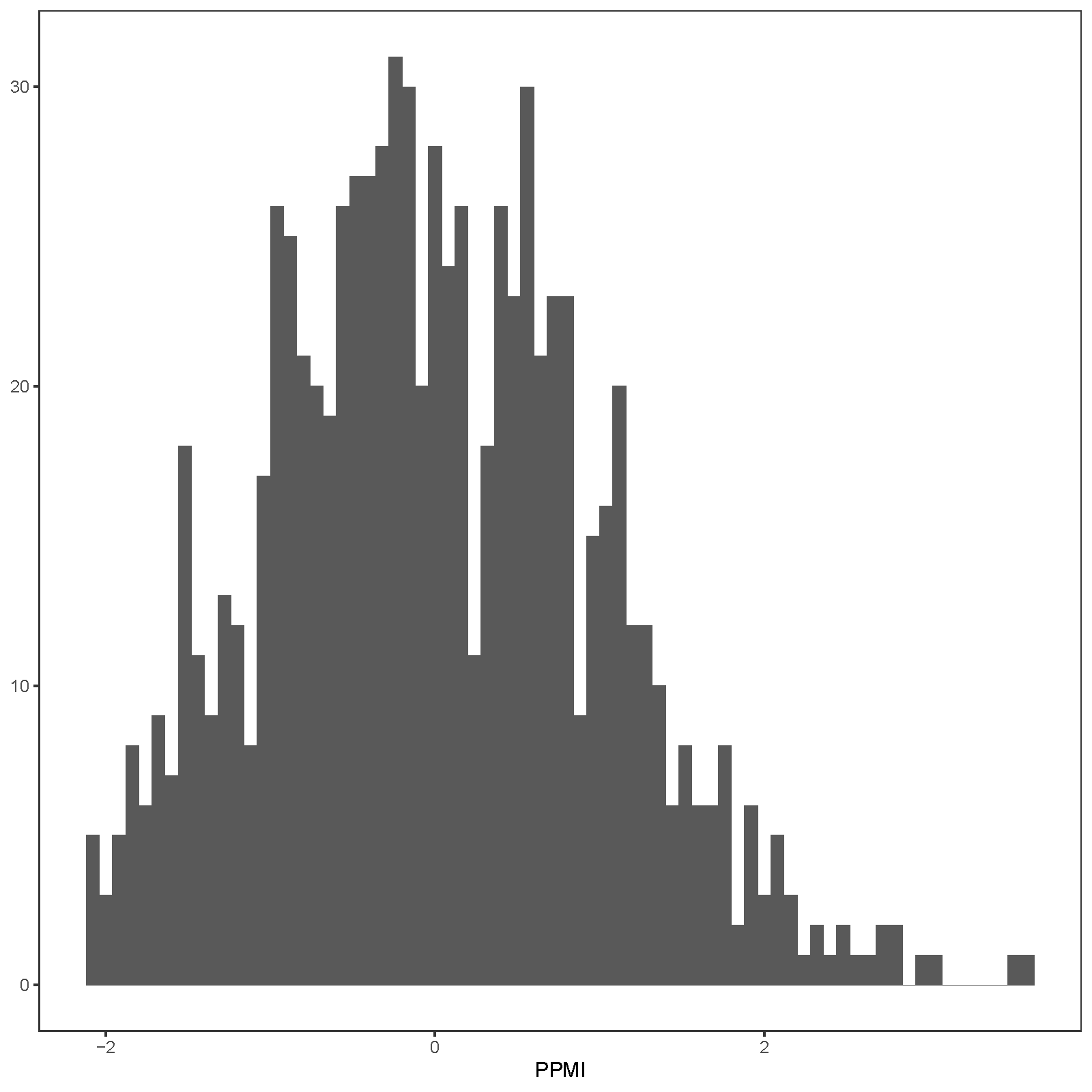


Figure S8. The distributions of T-tau (Standardized data).


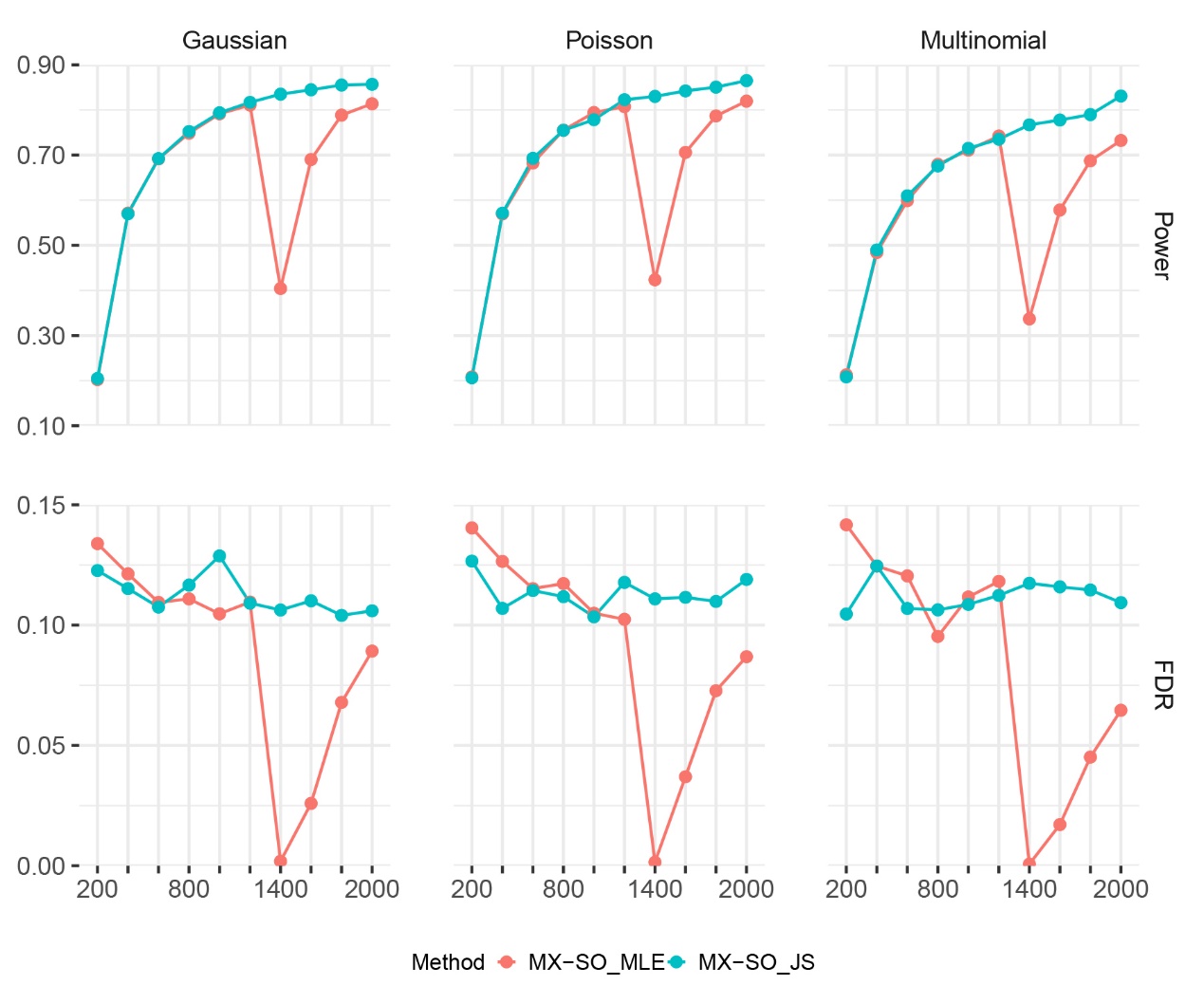


Figure S9. The power and FDP performances given different number of samples when covariance is estimated using the traditional maximum likelihood estimator (denoted as MX-SO_MLE) and the James-Stein-type shrinkage estimator (denoted as MX-SO_JS).
